# Ending the mass criminalisation of people who use drugs: a necessary component of the public health response to hepatitis C

**DOI:** 10.1186/1471-2334-14-S6-S4

**Published:** 2014-09-19

**Authors:** Chris Ford, Juliet Bressan

**Affiliations:** 1 IDHDP, Camden, London, UK; 2 General practitioner and Public Health Doctor, Health Service Executive, Ireland; 3 Addiction Services, Dublin North, Ireland

## 

There is clear evidence that the global HIV epidemic is fuelled by the war on drugs and by the criminalisation of people who inject drugs (PWID) [1]. We also have some evidence that the spread of hepatitis C virus (HCV) is fuelled in the same way [2], with the resulting stigmatisation and discrimination serving as barriers to HCV care and treatment.

Hepatitis C, like HIV, is preventable and treatable. Unlike HIV, it is curable. Yet it remains a major cause of morbidity and mortality, particularly in PWID [3]. There is much higher prevalence of HCV than HIV among PWID, especially in prisons. Global prevalence of HCV is estimated to be about 80% among PWID, versus a prevalence of roughly 2%–3% in general populations [4]. A staggering 90% of people who have been injecting drugs for more than 10 years are HCV-positive, as are half of people who have been injecting drugs for less than 10 years [2].

All health outcomes for PWID, including those associated with hepatitis C, are far worse in countries where a criminalisation approach is heavily favoured over the provision of drug treatment and other health services**.** Countries that respond to injecting drug use with a balanced policy have better health outcomes [5].

The emphasis in many countries on the mass incarceration of drug users is putting prisons at the centre of the hepatitis C epidemic. Between 16 and 41 percent of incarcerated people have HCV, and between 29%–43% percent of people with HCV have been in a correction facility [6]. Despite the widespread need, treatment and care of HCV-infected people in prisons is severely lacking.

Although HCV is the leading transmissible infection among PWID, the medical and drug policy communities have done little to address the HCV prevention and treatment needs of this population. The absence of attention to HCV in international drug policy development [3] has contributed to HCV becoming a global public health crisis.

The purpose of this commentary is to show how HCV has largely been ignored in drug prevention and treatment policies and to explain why ending the mass criminalisation of people who use drugs is a necessary component of the public health response to hepatitis C. The following section discusses barriers to an effective HCV response in PWID with emphasis on the criminalisation of PWID. It is followed by a section on overcoming barriers. The paper then concludes by calling for a shift from criminalisation to health promotion for PWID.

## Barriers to effectively addressing the HCV-related needs of people who inject drugs

The following all limit efforts to reduce HCV transmission among PWID and to improve health outcomes for those who become infected.

### Lack of advocacy

In contrast to the widespread and sustained advocacy movement that has come to characterise the global response to HIV, efforts to represent the interests of communities threatened by HCV are sporadic and fragmented. There is insufficient public and health provider awareness and support for preventing and controlling the disease. This is partly related to the fact that the main transmission route for HCV in many countries is injecting drug use, which is associated with prejudice and criminalisation. It is also difficult to galvanise a strong advocacy response because the low incidence of sexual transmission of HCV means that it is not perceived as a risk to the wider community.

### Lack of awareness in drug-using communities

Because HCV is about ten times more contagious than HIV, there is enormous risk from sharing injecting equipment. Many people who inject are unaware of or underestimate the riskiness of their own injecting behaviours. Many PWID who are infected with hepatitis C lack knowledge about its long-term health consequences. What knowledge they possess is often based on myths, outdated information, and conflicting messages from health providers.

### Inadequate testing

Although people who use drugs and incarcerated people are both high-risk groups, the levels of testing in these groups are poor [1].

### Lack of funding

A lack of funding for HCV prevention, treatment and research in PWID populations can be observed in many countries. Taking the United States as an example, at least five million people in the United States have been infected with HCV. That is about five times as many people as are infected with HIV, hence a public health response to the HCV epidemic is needed with appropriate investment in prevention, testing, treatment and research. However the US government has all but ignored the threat of HCV [7]. This may be linked to the difficulty of getting decision-makers to allocate public funding to a disease associated with injection drug use.

Treatment worldwide is costly, and the notoriously high cost of the newest HCV drugs is likely to further amplify concerns in this realm.

### The effects of stigma on health-seeking behaviour

Negative attitudes toward PWID encourage people who need health services to maintain hidden identities and to conceal behaviours that put them at risk for HCV. They may not want to acknowledge to general practitioners, infectious disease specialists, hepatologists and other health care providers that they have committed the “crime” of using drugs.

### Lack of health system and health professional responsiveness to PWID

Numerous health system-related factors help to determine whether or not PWID receive appropriate HCV services, including knowledge and attitudes of health care workers; the ease with which services facilitate anonymity and discretion; and practitioners’ ability to talk authentically and in necessary detail about someone’s behaviour and to recognize the day-to-day contingencies that apply for someone with a regular habit, who has inadequate means to fund it, and little social capital to bring to bear on the problem [8]. Furthermore, health systems often have poor and uncoordinated HCV treatment policies for PWID, with complex treatment pathways that are not linked to other components of health care such as addiction treatment.

Prejudice by treating physicians is a particular problem. Often PWID are thought to be unworthy of receiving treatment because they are viewed as criminals who brought the disease on themselves. A survey of Canadian physicians found that only 20% of HCV specialists would consider treating HCV in PWID [9]. In England there are huge variations in hepatitis C services offered to PWID across the country. For example, 10 hospitals refuse National Institute for Health and Clinical Excellence-approved treatment to all injecting drug users; 12 hospitals do not offer re-treatment to any patients, 55 offer it to some patients but with varying criteria; and two hospitals refuse treatment to anyone continuing to consume alcohol. Around one-third of hepatitis C patients referred to hospitals are not being offered treatment [10].

### Failure to scale up harm reduction strategies

Since the 1990s, health prevention and health promotion strategies aimed at PWID have emphasized harm reduction strategies for HIV prevention and treatment [11,12]. Such strategies have proven successful in reducing the prevalence of HIV in settings where they have been used [13]. Globally, however, the provision of HCV prevention and treatment services for PWID, such as needle and syringe programmes (NSPs), remains inadequate.

A worldwide review of coverage of NSPs in 2012 found that although 86 countries provide NSPs and 77 countries provide opiate substitution treatment (OST), rates of provision vary widely and do not relate to infectious disease rates or levels of need [14]. In some countries, political pressure is brought to bear on government officials to discourage them from providing public services to an “undeserving” sector of society.

In many countries where HCV is endemic, NSP availability is abysmally low. The situation is even worse for OST, which is an effective evidence-based treatment for drug dependence and also an effective form of HCV prevention. Only about 40% of countries provide OST, many inadequately [13]. In many countries, police oppression and other forms of structural violence further undermine efforts to deliver NSP and OST services. Most countries do not have NSPs in prisons and are reluctant to develop this service for prisoners due to concerns regarding cost, violence and custodial administrative issues [14].

### Incarceration of PWID and introduction of risks to wider prison populations

Incarceration is an independent risk factor for HCV, [15] and many of the groups at highest risk for HCV infection in the community remain at risk in prison. (Women in prison are more likely to have HCV than their male counterparts, unlike in the general population [16].) Many people initiate injecting drug use in prison [11]. Furthermore, custodial facilities have poor disease surveillance systems.

Custodial facilities expose “at risk” young people to injecting drugs, which they may not have accessed in their communities. Prisoners may also have existing risk factors for injecting, which leads to increased HCV risk, such as psychiatric disease, while also taking on other risks in prison, such as alienation and boredom. The culture in prisons can lead to additional risks for HCV transmission including tattooing, barbering, fighting, contact sports and rape. Prison officers can discourage the adoption of protective behaviours, for example when prisoners’ requests for bleach lead to searches and further punishment [14].

## Recommendations for overcoming barriers

Although there are formidable barriers to HCV prevention and treatment for PWID, much can be done to overcome these barriers. Over the past few years, the seeds of HCV advocacy groups around the world have begun to make a difference. These include patient support groups to help people through treatment as well as broadly focused national organisations such as the Hepatitis C Trust in the United Kingdom and globally the World Hepatitis Alliance. Also, there are increasingly more examples of accessible information about HCV circulating in PWID communities.

However, significant progress will not be achieved without changes at the national level in many countries. Policy-makers and all stakeholders must become more cognisant of the public health magnitude of the HCV threat and the cost-effectiveness of funding HCV interventions for PWID. The health costs of incarceration need to be addressed, including showing that treating hepatitis C in the prison population is cost-saving [17].

It is essential to fight stigma and to correct false information. The misconception that addiction is a moral issue must be challenged, and barriers that prevent patients from accessing information and services must be removed. Strategies for overcoming some key barriers are highlighted below.

### Improve HCV testing for PWID

HCV testing has improved in some countries, notably in European countries and in Australia, but efforts can be better targeted at most-at-risk groups such as people who use drugs and people who are in prison [18,19]. The organisation of services needs to be considered, since HCV testing has improved most when it has been integrated into existing facilities such as addiction services and general practices.

### Improve access to care and treatment for PWID

Increased funding and access for PWID will help with prevention and with access to care and treatment, but this funding needs to be used in a coordinated strategic way [20,21], as the example of Scotland demonstrates. In 2000, a report by the Scottish Needs Assessment Programme (SNAP) prompted the Scottish Government to recognize that Hepatitis C was one of the most serious and significant public health risks, and to set about dealing with HCV in a strategic way. The SNAP report brought together existing initiatives to tackle hepatitis C and made recommendations on how prevention, diagnosis and treatment could be improved. An action plan was designed to implement these recommendations, and the key messages emerged in April 2004 [22]. The three principle objectives were to reduce the transmission of HCV among current PWID; to diagnose infected persons, particularly those who are most in need of therapy; and to provide optimal care and support for HCV-diagnosed persons. The action plan was funded and coordinated appropriately including a national database of diagnoses; public awareness-raising; national needle exchange surveys; training for staff at all levels; and models of best practice. HCV came to be viewed as a disease that the country needed to address rather than being marginalised as a problem for the PWID community [22].

### Provide and improve needle and syringe programmes and opiate substitution treatment services

With some reservations, we have long known that the availability of NSPs and OST can reduce the prevalence of HCV [13]. Scaling up OST and high-coverage needle and syringe programmes can reduce hepatitis C prevalence among PWID, but reductions can be modest and require long-term sustained intervention coverage. In high-coverage settings, other interventions are needed to further decrease hepatitis C prevalence. In low-coverage settings, sustained scale-up of both interventions is needed [23]. Neither intervention protects as effectively against HCV as it does against HIV because HCV is a more robust virus with greater transmissibility. Prevalence of HCV is already high in many populations by the time NSPs and OST become available, which means that high levels of coverage of both interventions need to be maintained on a long-term basis, in the community as well as in prisons.

### Training for people who interact with PWID

Training on HCV and harm reduction needs to be provided to doctors, nurses and other health professionals, and this training needs to encompass the stigma and discrimination associated with drug use. Ancillary and prison staff need to be trained to provide quality information about harm reduction and HCV to at-risk groups.

## Conclusion: shifting the focus from criminalisation to health promotion

The most important principle for improving the response to HCV among people who inject drugs is that drug policy and drug treatment decisions need to be based on health objectives. When at-risk individuals come to judicial attention, they should be offered health interventions rather than incarcerated and marginalised.

Police oppression, harmful drug policies and other forms of structural violence need to be addressed. In parts of Southeast Asia and the former Soviet Union, barriers would be greatly reduced if existing laws were simply implemented less brutally.

Building integrated drug dependence treatment systems that bring together stakeholders from drug prevention, health and law enforcement fields is essential. There is a need for initiatives that aim to link these spheres, such as the Centre for Law Enforcement and Public Health. This web-based programme run from Australia is committed to pursuing projects and advancing knowledge in the joint fields of policing and other law enforcement and the many aspects of public health [24].

One of the most important actions that must be taken is the amendment of laws that criminalise, stigmatise and marginalise people who use drugs. Where this approach was tried, such as in Portugal, the number of street overdoses fell, illicit drug use dropped, and there was a reduction in HIV prevalence among PWID [25]. Changes in HCV trends were not monitored, but it is reasonable to speculate that positive outcomes may have occurred in this regard as well.

The human and societal costs of the HCV crisis must be recognised. Governments must realise that in some ways HCV has a similar public health impact as HIV. They should address all of the policy issues mentioned above, including lack of funding and uncoordinated strategies. Providing adequate and coordinated country-wide programmes in the community and in prisons saves money and lives, as does providing access to treatment for people who use drugs.

It is time to shift away from arresting and incarcerating people for using drugs, and to instead focus on their health – which includes acting to prevent more people from dying unnecessarily from HCV.

## Competing interests

The authors declare that they have no competing interests.

## Declarations

This article has been published as part of *BMC Infectious Diseases* Volume 14 Supplement 6, 2014: Viral Hepatitis in Europe. The full contents of the supplement are available online at http://www.biomedcentral.com/bmcinfectdis/supplements/14/S6. The publication charges for this supplement were funded by AbbVie as an unrestricted grant to Rigshospitalet, the University of Copenhagen. AbbVie further funded the printing of the supplement with additional financial support from the Drug Prevention and Information Programme (DPIP) of the European Union.
